# Expression Signatures of Metastatic Capacity in a Genetic Mouse Model of Lung Adenocarcinoma

**DOI:** 10.1371/journal.pone.0005401

**Published:** 2009-04-30

**Authors:** Don L. Gibbons, Wei Lin, Chad J. Creighton, Shuling Zheng, Dror Berel, Yanan Yang, Maria Gabriela Raso, Diane D. Liu, Ignacio I. Wistuba, Guillermina Lozano, Jonathan M. Kurie

**Affiliations:** 1 Department of Thoracic/Head and Neck Medical Oncology, The University of Texas - M. D. Anderson Cancer Center, Houston, Texas, United States of America; 2 Dan L. Duncan Cancer Center, Baylor College of Medicine, Houston, Texas, United States of America; 3 Department of Molecular Genetics, The University of Texas - M. D. Anderson Cancer Center, Houston, Texas, United States of America; 4 Department of Pathology, The University of Texas - M. D. Anderson Cancer Center, Houston, Texas, United States of America; 5 Biostatistics and Applied Mathematics, The University of Texas - M. D. Anderson Cancer Center, Houston, Texas, United States of America; Ordway Research Institute, United States of America

## Abstract

**Background:**

Non-small cell lung cancer (NSCLC) is the foremost cause of cancer-related death in Western countries, which is due partly to the propensity of NSCLC cells to metastasize. The biologic basis for NSCLC metastasis is not well understood.

**Methodology/Principal Findings:**

Here we addressed this deficiency by transcriptionally profiling tumors from a genetic mouse model of human lung adenocarcinoma that develops metastatic disease owing to the expression of *K-ras^G12D^* and *p53^R172H^*. We identified 2,209 genes that were differentially expressed in distant metastases relative to matched lung tumors. Mining of publicly available data bases revealed this expression signature in a subset of NSCLC patients who had a poorer prognosis than those without the signature.

**Conclusions/Significance:**

These findings provide evidence that *K-ras^G12D^*; *p53^R172H^* mice recapitulate features of human NSCLC metastasis and will provide a useful platform on which to study the biologic basis for lung adenocarcinoma metastasis and its prevention by novel agents.

## Introduction

Non-small cell lung cancer (NSCLC) is the leading cause of cancer-related death in the United States and other western countries. Approximately two thirds of patients are diagnosed at an advanced stage, and of the remaining patients who undergo curative surgery, 30–50% have a recurrence with metastatic disease. Thus, a better understanding of the biologic underpinnings of metastatic disease is of paramount importance. Metastasis research in lung cancer has been hampered by the lack of good animal models and the difficulty in studying disease progression and metastasis in patients. This has resulted in a reliance on *in vitro* cell cultures derived from patients or immunodeficient animal xenograft studies. As a result, we understand much more about cancer cell-autonomous genetic and epigenetic changes than about the role of the supportive microenvironment. To address this need, we and other investigators have developed mouse models in which lung adenocarcinomas arise spontaneously owing to mutant *K-ras* alleles expressed inducibly, conditionally, or somatically [1], [2], [3], [4], [5]. Although an improvement, these models uniformly lack metastatic potential, a serious deficiency given that metastasis is the most common cause of death in NSCLC patients.

A *p53* missense mutation, R175H, found in Li-Fraumeni syndrome patients and in a subset of NSCLC patients, is a structural mutation that exhibits loss of function owing to inactivation of p53 transcriptional activity [6], [7], [8]. Mutation of the corresponding arginine (R172H) in murine *p53* has been previously introduced into the mouse as a knock-in allele. To evaluate the importance of p53^R172H^ as a contributing event in lung tumorigenesis, p53^R172HΔG^ mice were previously mated with Kras^LA1^ mice, which develop lung adenocarcinomas owing to somatic activation of a latent Kras^G12D^ allele, but rarely metastasize [4]. Mice were generated that were heterozygous for Kras^LA1^ alone, p53^R172HΔG^ alone, or both alleles [9]. In the absence of mutant *K-ras*, lung adenocarcinomas were rare (13% of p53^R172HΔG/+^ mice). Although the presence or absence of the mutant *p53* allele did not affect the frequency of lung adenocarcinomas in Kras^LA1^ mice (62.5% *versus* 70.8%, respectively), metastases were much more frequent in those with *p53* mutations than in those without (36.5% *versus* 4.5%). In the Kras^LA1/+^; p53^R172HΔG/+^ mice, metastases were found at sites frequently observed in NSCLC patients, including the mediastinal lymph nodes, heart, parietal pleura, diaphragm, liver, adrenal gland, kidney, mesentery, pancreas, and subcutaneous tissues. The remaining wild-type *p53* allele was deleted in approximately 50% of the murine tumors, which mimics the wild-type *p53* allelic deletion observed in tumors from Li-Fraumeni patients. Collectively, these findings suggest that Kras^LA1/+^; p53^R172HΔG/+^ mice are a useful model for the study of metastasis in NSCLC patients.

In this study, we sought to better understand the biologic basis for metastasis in Kras^LA1/+^; p53^R172HΔG/+^ mice. Tumors from the lung and distant metastatic sites were transcriptionally profiled, from which we derived a metastasis signature defined as those genes that were differentially expressed in the metastases relative to paired primary lung tumors. Data mining of publicly-available expression profiles revealed this signature in a subset of primary tumors from NSCLC patients who had poor prognosis. We conclude that Kras^LA1/+^; p53^R172HΔG/+^ mice are a useful tool for the study of lung adenocarcinoma metastasis.

## Materials and Methods

### Mouse studies

We followed the guidelines set forth by the Institutional Animal Care and Use Committee of The University of Texas, M. D. Anderson Cancer Center for husbandry of *p53^R172HΔg/+^ K-ras^LA1/+^* mice.

### Tumor samples

Primary lung adenocarcinomas and metastases from *p53^R172HΔg/+^ K-ras^LA1/+^* mice were isolated, carefully dissected to remove the adjacent tissue, snap-frozen in liquid nitrogen and stored at −80° until use [9]. Part of each dissected tumor was histologically evaluated by a board-certified pathologist.

### Gene expression profiling

Total RNA from the *p53^R172HΔg/+^ K-ras^LA1/+^* tumors was extracted by Trizol (Invitrogen) and purified with an RNeasy kit (Qiagen). RNA quality and quantity were evaluated on an Agilent Bioanalyzer following the manufacture's recommendations (Agilent Technologies). Synthesis of cRNA and hybridization to Mouse Expression Array 430A 2.0 chips were performed following Affymetrix protocols (Affymetrix, Inc.).

### Microarray data analysis

After scanning and low-level quantification using Microarray Suite (Affymetrix), DNA Chip (dChip) analyzer [10] was used to estimate expression values, using the PM/MM difference model and invariant set normalization. Present call rates for the tumor profiles ranged from 51% to 63%, and none of the profiles were flagged by dChip as potential outliers. Two-sided *t*-tests using log-transformed data determined significant differences in mean gene mRNA levels between groups of paired samples. Fold changes between groups were estimated by taking the averages of the metastasis/primary log ratios. Expression values were visualized as color maps using the Java TreeView software [11]. Gene Ontology (GO) annotation terms were searched within gene sets using SigTerms [12]. Genes arising from the syngenic tumor dataset were clustered using the technique described in [13]. Expression profiles were deposited into the Gene Expression Omnibus data repository (GSE accession #14449) and are MIAME compliant.

**Figure 1:**
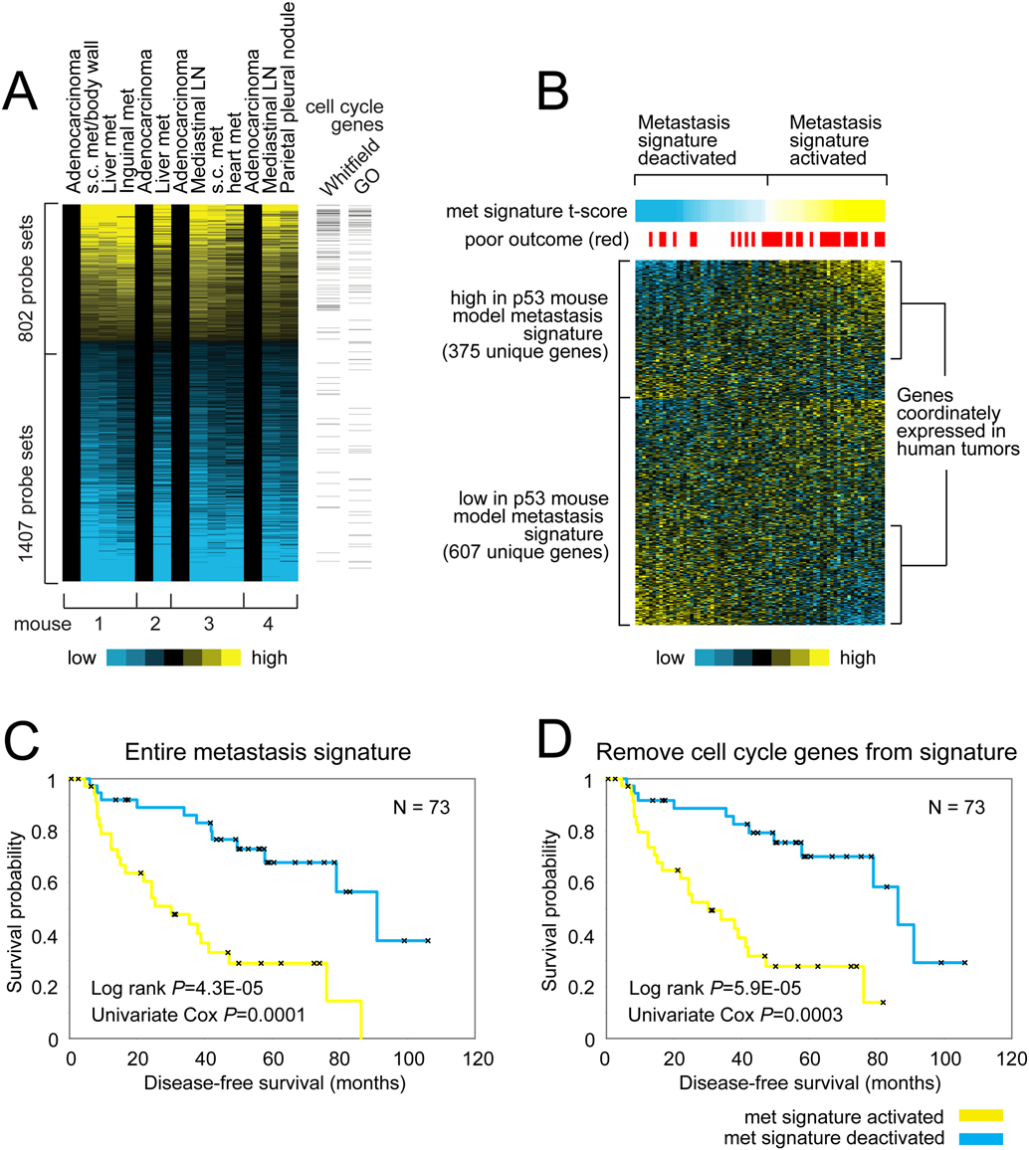
A gene expression signature of spontaneous metastasis in a K-ras/p53 mutant mouse model. (A) Gene expression profiles of tumor metastases were compared to the corresponding primary tumor to define the metastasis gene signature (P<0.01, paired t-test). Each row of the expression matrix represents a gene and each column represents a profiled sample; relative gene expression (metastasis: primary) is represented using a yellow–blue color scale. Genes defined as cell cycle-related by either the Whitfield signature [16] or by Gene Ontology (GO) are indicated. (B) The expression patterns of the mouse model metastasis signature in a panel of human lung tumors from Bhattacharjee et al. [14]. Tumors showing “activation” of the metastasis signature (as measured by the “met signature t-score”) tend to have high expression of the genes high in the mouse metastases and low expression of the genes low in the mouse metastases. (C) Kaplan-Meier analysis of the human lung tumors comparing the differences in risk between tumors showing activation (yellow line, t-score>0) and tumors showing deactivation (blue line, t-score<0) of the mouse model metastasis signature. Log rank test evaluates whether there are significant differences between the two arms. Univariate Cox test evaluates the association of the met signature t-score with patient outcome, treating the coefficient as a continuous variable. (D) Same as for part C, except that cell cycle-associated genes (as defined by either Whitfield et al or GO) were first removed from the mouse model metastasis signature prior to deriving the met signature t-score.

In order to score each human lung tumor within a set for similarity to our gene signature of spontaneous metastases (Figure 1), we derived a “t-score” for each human tumor in relation to the mouse metastasis signature, similar to what we have done in previous analyses [13]. The t-score was defined as the Pearson's correlation between the mouse metastasis gene signature pattern (using “1” and “−1”, for up and down, respectively) and the human tumor's expression values (which is essentially a t-statistic comparing the average of the up genes with that of the down genes within each human tumor). The gene expression values in the human tumor datasets were first normalized to standard deviations from the mean before computing the t-score. The mapping of transcripts or genes between the mouse signature and the human tumor array datasets was made on the Entrez Gene identifier; where multiple human array probe sets referenced the same gene, the probe set with the highest variation represented the gene.

## Results

### Transcriptional profiling of spontaneous tumors from Kras^LA1/+^; p53^R172HΔG/+^ mice reveals a metastasis signature that is prognostic in NSCLC patients

**Figure 2:**
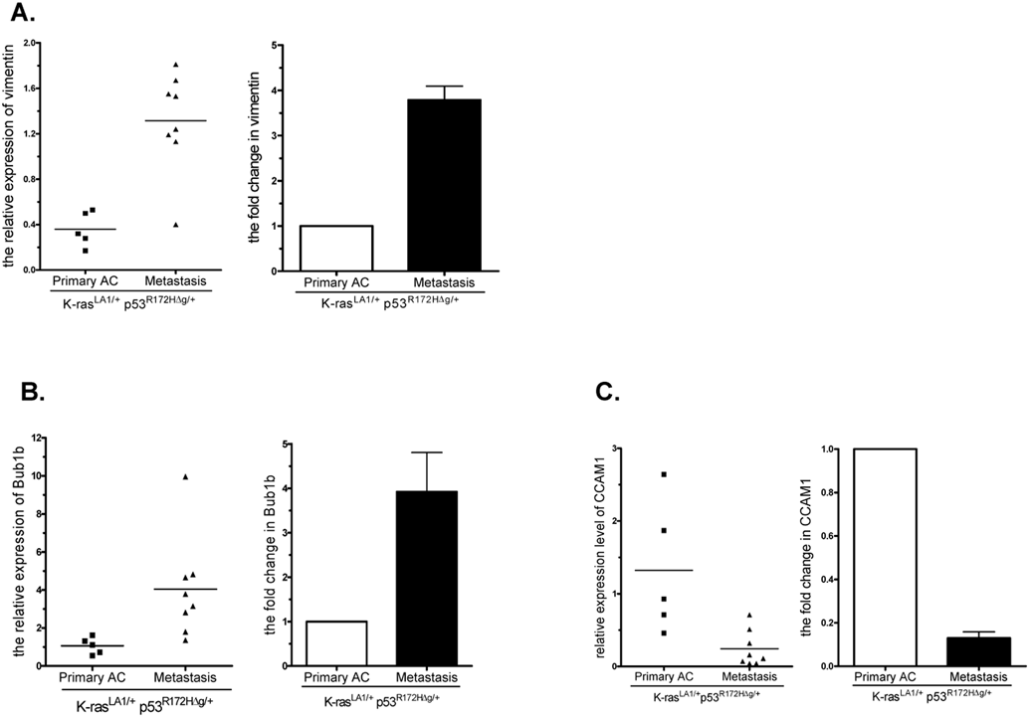
Verification of mRNA expression levels by Q-PCR. Total RNA from spontaneous tumors for gene expression profiling was reverse-transcribed with the First-Strand cDNA Synthesis Kit (Amersham Bioscience). Real-time PCR reactions were prepared in duplicate in a 96- or 384-well clear optical reaction plate (Applied Biosystems), using SybrGreen master mix (Applied Biosystems), and run on an ABI PRISM 7900HT Sequence Detection System (Applied Biosystems). Normal lung tissue from wild-type mice was used for calibration. Glyceraldhyde-3-phosphate dehydrogenase (GAPDH) was used as the endogenous control. The relative level of a gene was determined by calculating ΔΔCt, based on the formula ΔΔCt = (sample Ct [gene]−sample Ct [GAPDH])−(normal lung Ct [gene]−normal lung Ct [GAPDH]).

We postulated that the biologic processes mediating metastasis in Kras^LA1/+^; p53^R172HΔG/+^ mice would recapitulate those in a subset of NSCLC patients. To test this, the transcriptome of tumors from Kras^LA1/+^; p53^R172HΔG/+^ mice were profiled, and the derived metastasis signature, which was defined as those genes that were differentially expressed in the metastases relative to paired primary lung tumors, was compared to previously published expression profiles from lung adenocarcinomas from several patient cohorts [14], [15]. Primary lung and matched metastastic tumor tissues from 4 mice were removed (Table 1); RNA was purified and subjected to Affymetrix gene expression profiling. Using each primary lung tumor (n = 4, 1 per mouse) as the reference for the corresponding metastases (n = 9, 1 to 3 per mouse), 2,209 genes were found to be differentially expressed (p value<0.01, paired t-test), 802 of which were increased and 1,407 were decreased (Figure 1A). Listed in Table 2 and Figure S1 are the most over- and under-expressed genes in the metastases and the entire set of 2,209 differentially expressed genes, respectively. We validated differential expression of genes involved in processes relevant to metastasis, including BUB-1, a regulator of genomic integrity and mitosis, VIM, a marker of epithelial-to-mesenchymal transition, and the adhesion molecule, CCAM1, by performing quantitative reverse transcriptase PCR analysis (Figure 2).

**Table 1:** Tumors Used for Gene Expression Analysis.

Genotype	Mouse #	Tumor type and location
p53R172HΔg/+ K-rasLA1/+	mouse 1	AC18-adenocarcinoma, lung
		AC19-metastasis, liver
		AC20-metastasis, body wall 1
		AC21-metastasis, body wall 2
p53R172HΔg/+ K-rasLA1/+	mouse 2	AC22-adenocarcinoma, lung
		AC23-metastasis, liver
p53R172HΔg/+ K-rasLA1/+	mouse 3	AC24-adenocarcinoma, lung
		AC25-metastasis, heart
		AC26-metastasis, mediastinal lymph node
		AC27-metastasis, body wall
p53R172HΔg/+ K-rasLA1/+	mouse 4	AC31-adenocarcinoma, lung
		AC32-metastasis, mediastinal lymph node
		AC33-metastasis, parietal pleura

**Table 2:** Top named genes differentially expressed (*P*<0.01) between primary tumors and metastasis.

Affymetrix probe set	Gene Title	Gene Symbol	fold change, met vs primary (log2)
*Higher in metastasis (ranked by fold change)*			
1426278_at	interferon, alpha-inducible protein 27	Ifi27	2.034805214
1418588_at	neurensin 1	Nrsn1	2.015696907
1423439_at	phosphoenolpyruvate carboxykinase 1, cytosolic	Pck1	2.008413051
1436504_x_at	apolipoprotein A-IV	Apoa4	1.899587267
1448226_at	ribonucleotide reductase M2	Rrm2	1.856439628
1427465_at	ATPase, Na+/K+ transporting, alpha 2 polypeptide	Atp1a2	1.80166054
1419943_s_at	cyclin B1	Ccnb1	1.718304491
1460347_at	keratin 14	Krt14	1.650172294
1438009_at	similar to histone 2a	MGC73635	1.628484792
1455439_a_at	lectin, galactose binding, soluble 1	Lgals1	1.619253423
1431164_at	Ras-related GTP binding D	Rragd	1.616979652
1451367_at	COP9 (constitutive photomorphogenic) homolog, subunit 6 (Arabidopsis thaliana)	Cops6	1.616770969
1426920_x_at	integrin beta 1 (fibronectin receptor beta)	Itgb1	1.607222931
1416301_a_at	early B-cell factor 1	Ebf1	1.604511281
1422006_at	eukaryotic translation initiation factor 2-alpha kinase 2	Eif2ak2	1.584624642
1448314_at	cell division cycle 2 homolog A (S. pombe)	Cdc2a	1.564221952
1420575_at	metallothionein 3	Mt3	1.543842484
1419513_a_at	ect2 oncogene	Ect2	1.525512151
1456566_x_at	RNA binding motif protein 14	Rbm14	1.496890793
1423607_at	lumican	Lum	1.4854932
*Lower in metastasis (ranked by fold change)*			
1452543_a_at	secretoglobin, family 1A, member 1 (uteroglobin)	Scgb1a1	−5.605051087
1435386_at	Von Willebrand factor homolog	Vwf	−4.501907686
1423436_at	glutathione S-transferase, alpha 3	Gsta3	−3.815193669
1421802_at	eosinophil-associated, ribonuclease A family, member 1	Ear1	−3.81250599
1454681_at	RNA binding motif protein 35A	Rbm35a	−3.599463463
1416236_a_at	epithelial V-like antigen 1	Eva1	−3.579430627
1422905_s_at	flavin containing monooxygenase 2	Fmo2	−3.576820392
1419475_a_at	ets homologous factor	Ehf	−3.487510424
1450494_x_at	CEA-related cell adhesion molecule 1	Ceacam1	−3.394496
1423914_at	RIKEN cDNA C630004H02 gene	C630004H02Rik	−3.049771708
1423323_at	tumor-associated calcium signal transducer 2	Tacstd2	−3.037340132
1429626_at	surfactant associated protein A1	Sftpa1	−2.893831126
1449081_at	carboxylesterase 3	Ces3	−2.88430922
1426332_a_at	claudin 3	Cldn3	−2.869993162
1422334_a_at	surfactant associated protein A1	Sftpa1	−2.7988671
1417797_a_at	RIKEN cDNA 1810019J16 gene	1810019J16Rik	−2.785446923
1449184_at	peptidoglycan recognition protein 1	Pglyrp1	−2.713447175
1418639_at	surfactant associated protein C	Sftpc	−2.710279321
1417275_at	myelin and lymphocyte protein, T-cell differentiation protein	Mal	−2.707840748
1421404_at	chemokine (C-X-C motif) ligand 15	Cxcl15	−2.682133804

Enrichment analysis (Fisher's exact test using Gene Ontology terms) of those genes with increased expression revealed highly significant enrichment in genes with the terms ‘cell cycle’ (45 genes, enrichment p = 1.4E-9), ‘kinetochore’ (8 genes, p = 1.5E-6), ‘pericentric chromosome-binding’ (10 genes, p = 3.4E-6), ‘DNA replication’ (17 genes, p = 6.0E-6), and ‘DNA-binding’ (103 genes, p = 0.0001), whereas analysis of the genes that were decreased revealed enrichment in genes with the terms ‘membrane-binding’ (391 genes, p = 9.8E-12), ‘integral-to-membrane’ (333 genes, p = 5.1E-9), ‘lysosomal’ (24 genes, p = 1.2E-5), and ‘golgi apparatus’ (29 genes, p = 0.0001) (a complete list of the terms is in Figure S2). A significant number of genes appeared to be related to cell cycle functions (Fig. 1A), as defined by the Gene Ontology classification or using the signature from Whitfield et al. [16].

We next compared these results with a publicly-available database containing expression profiles of resected, early-stage NSCLC specimens from the dataset by Bhattacharjee et al. [14], for which clinical outcome data was available. Focusing the analysis on those patients with lung adenocarcinomas (n = 73), we examined whether the murine metastasis signature is present in patients and whether its presence correlates with poor clinical outcome, which would be expected if the signature indicates the presence of tumor cells with the capacity to metastasize. Of the 1,407 genes with differential expression in the murine metastasis signature, 982 (70%) genes were represented in the human tumor expression profiles. Each human lung tumor was assigned a metastasis t-score, which gave a measure of how the human tumor recapitulated the patterns of over- and under-expression observed in the murine metastasis signature (Fig. 1B). Using this approach, we found that the level of enrichment of the Bhattacharjee tumors for the murine metastasis signature was informative from a prognostic standpoint, whether or not the genes related to cell cycle were included (Fig. 1C and 1D). Those patients with the signature (t-score>0) had a shorter median disease-free survival duration than did those without the signature (p<0.001, Kaplan-Meier analysis) (Fig. 1C).

**Figure 3:**
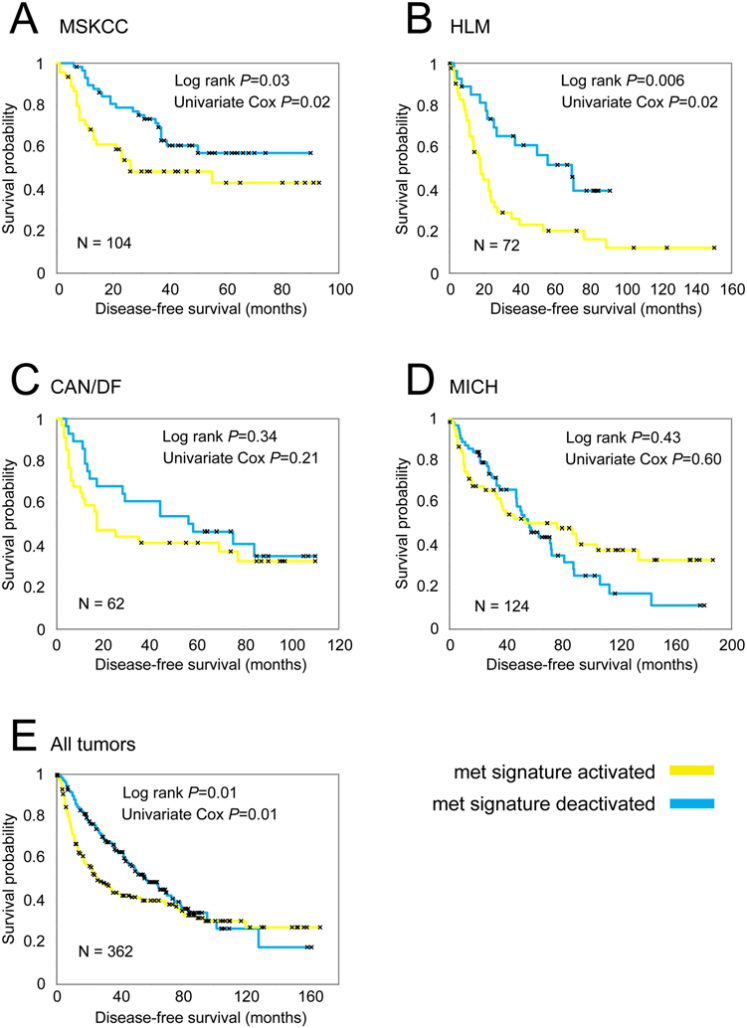
The spontaneous mouse metastasis signature is associated with poor prognosis in human lung tumor profile datasets from the Director's Challenge Consortium. Kaplan-Meier analysis comparing the differences in risk between human lung tumors showing activation (yellow line, t-score>0) and tumors showing deactivation (blue line, t-score<0) of the mouse model metastasis signature. Datasets from the study by Shedden et al. [15] and represent four independent cohorts from (A) Memorial Sloan-Kettering Cancer Center (MSK), (B) Moffitt Cancer Center (HLM), (C) Dana-Farber Cancer Institute (CAN/DF), and (D) University of Michigan Cancer Center (MICH). (E) Kaplan-Meier analysis of tumors combined from all four datasets (N = 362).

Using the same parameters and methodology, we examined the four human NSCLC datasets presented in the Director's Challenge study [15] and found that, for two of the cohorts (MSKCC and HLM) in the Director's Challenge study, those patients with tumors that had the metastasis signature (t-score>0) had a shorter progression-free survival than did those without the signature (p< = 0.03 for each, Kaplan-Meier analysis, Fig. 3A & B). This prognostic trend was apparent in the Dana-Farber Cancer Institute (CAN/DF) cohort, though not with statistical significance (p = 0.13, Fig. 3C). The Michigan cohort, however, did not show any prognostic trend for the mouse metastasis signature (Fig. 3D), though among the 395 genes that were increased in the murine metastasis signature and represented on the Michigan arrays, 141 genes correlated (p<0.01, t-test) with poorly-differentiated versus well-differentiated histology (enrichment p value<1.0×10^−15^). An overall analysis of the four datasets combined did demonstrate significant prognostic ability in the human tumors by the murine metastasis signature (p = 0.01, Fig. 3E). Essentially the same results were obtained when the murine signature without the cell cycle genes was applied to the datasets (Figure S3). On the basis of these findings, we conclude that the murine model recapitulated biologic features of the subset of NSCLC adenocarcinoma patients with poor clinical outcomes.

To compare the metastasis signatures in Kras^LA1/+^; p53^R172HΔG/+^ mice to genes that have been implicated in metastasis of human tumors, we examined whether the murine signature overlapped with gene expression profiles of human primary *versus* metastatic tumor specimens [17], [18], which revealed a significant degree of overlap (P value<0.05, described in detail in Figure S4). Of the 738 genes that were increased (p<0.05), in the human metastasis relative to that of primary tumor, 49 were among the increased expression gene set in the murine signature (enrichment *p* = 0.001).

## Discussion

In this study, we sought to examine the fidelity of the metastatic process in *Kras^LA1/+^*; *p53^R172HΔG/+^* mice to that of NSCLC patients by performing transcriptional profiling studies. We identified a metastasis expression signature in *Kras^LA1/+^*; *p53^R172HΔG/+^* mice that was present in primary tumors from NSCLC patients who had poor prognosis. Based upon the ability of the murine metastasis signature to discriminate patient outcome, we conclude that the murine tumors recapitulated features of human lung adenocarcinoma. We do not mean to imply that this signature is clinically useful in a prognostic or predictive fashion, but simply interpret it as evidence of the potential usefulness and relevance of the model for studying the biology of human lung adenocarcinoma metastasis.

We examined whether the murine metastasis signature overlapped with genes identified from five NSCLC patient cohorts reported in two studies [14], [15] and found overlap in a subset of patients. The presence of the murine signature correlated with poor clinical outcome in only three of the five cohorts. We can only speculate about why the correlation with clinical outcome differed among the cohorts but suspect that it relates to tumor biologic differences. Tumor histology and disease stage are unlikely to be relevant variables because the distributions of these variables did not differ significantly between the cohorts examined, but patient demographic variables yet to be examined might prove relevant. The two NSCLC cohorts we used for data mining have reported both overall survival and disease progression-free survival [14], [15]. Although the murine metastasis signature identified patients with a poor clinical outcome with both clinical outcomes, trends were more significant with progression-free survival (Figure S3), implying that the genes in the murine signature impact biologic processes involved in disease recurrence but not other processes relevant to the survival of patients with recurrent disease, such as resistance to cancer treatments. Of note, the murine metastasis signature did not correlate with NSCLC *K-ras* mutational status, which was reported by Bhattacharjee et al. [14].

The increased-expression gene set in the metastasis signature was enriched in cell cycle genes, but the prognostic power of the metastasis signature was not diminished by the removal of cell cycle genes, suggesting that the metastatic capacity of lung tumor cells in *Kras^LA1/+^*; *p53^R172HΔG/+^* mice was not related simply to their proliferative potential and that other genes involved in biologic processes relevant to metastasis, such as tumor cell invasive potential, might have contributed. Of note, in that regard, were genes in the decreased-expression gene set that control cell polarity (*Cldn3*, *Pard3*, *Pard6b*, *Dlgh1*, and *Crb3*) and cell-cell attachments (*Ccam1* and *Cask2*). Loss of polarity and cell-cell contacts are features of epithelial cells that have undergone epithelial-to-mesenchymal transition (EMT), a phenotypic change associated with enhanced invasive and metastatic properties in tumor cells [19]. Other genes typically expressed in mesenchymal cells, including *Vim* and *Cdh2*, were more highly expressed in the metastases than in the primary lung tumors that arose in *K-ras^LA/+^ p53^R172HΔg/+^* mice. Whether these changes reflect a phenotypic change that contributed to the metastatic capacity of these cells is currently under investigation and will be reported on separately.

We conclude that *Kras^LA1/+^*; *p53^R172HΔG^* mice will provide a useful platform to better understand the basic biologic processes that underlie metastasis, to identify biologic targets for the prevention and treatment of metastasis, and to test the efficacy of novel agents directed against those targets in preclinical studies. Such studies could have a tremendous impact on global health given that NSCLC is the most common cause of cancer-related death in Western countries, and metastasis is the most common cause of death in NSCLC patients.

## Supporting Information

Figure S1 Excel spreadsheet with entire list of genes.
(0.74 MB XLS)

Figure S2 Excel spreadsheet with list of GO terms
(1.03 MB XLS)

Figure S3 PDF describing “Impact of cell cycle genes on progression-free survival and overall survival”
(0.08 MB PDF)

Figure S4 PDF describing “Comparison of mouse metastasis signature with other metastasis signatures”
(0.27 MB PDF)
