# Supplementary material for: Expression Signatures of Metastatic Capacity in a Genetic Mouse Model of Lung Adenocarcinoma
Source: PLoS One. 2009 Apr 30;4(4):e5401. doi: 10.1371/journal.pone.0005401 (PMC2671160; doi:10.1371/journal.pone.0005401)
Supplement: Figure S3 — PDF describing “Impact of cell cycle genes on progression-free survival and overall survival” (0.08 MB PDF) [file pone.0005401.s003.pdf]

Expression Signatures of Metastatic Capacity in a Genetic Mouse Model of Lung Adenocarcinoma

Supporting Document 1

(Impact of cell cycle genes on progression-free survival and overall survival)

Goal

- Examine prognostic ability of mouse metastasis signature in tumors from Bhattacharjee *et al*. and Shedden *et al*.
- As compared to the analysis presented in the main paper, carry out the following:
  - Remove cell cycle-associated genes from the mouse signature before carrying out the analysis
  - Consider “overall survival” as the endpoint in addition to “progression-free survival.”

Bhattacharjee dataset, overall survival

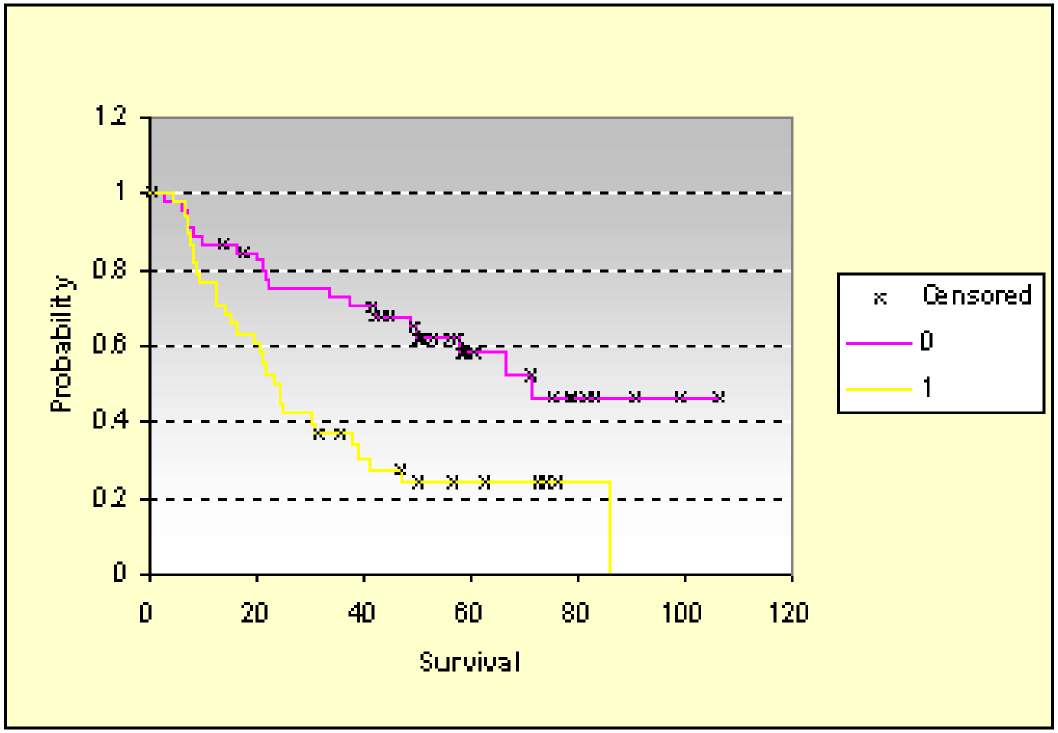

- Pink line=tumors with deactivated metastasis signature (R-value<0).
- Yellow line=tumors with activated metastasis signature (R-value>0).
- Difference between the two arms is significant (p<0.001, log-rank statistic).

Cell cycle genes in signature

- Genes up in mouse metastasis were significantly enriched for cell cycle genes.
- Is the association of the mouse met signature with outcome due to general proliferation?
- Carry out two tests:
  - Subtract genes with GO annotation "cell cycle" or correlated with cell cycle progression in Whitfield study from the met signature
    - Remaining genes are predictive in Bhattacharjee data with $p<0.001$ by univariate Cox.
  - Compute an R-value for the Whitfield cell cycle

Director’s Challenge datasets

Gene expression–based survival prediction in lung adenocarcinoma: a multi-site, blinded validation study

Director’s Challenge Consortium for the Molecular Classification of Lung Adenocarcinoma:*[1]
Kerby Shedden[2,3,17], Jeremy M G Taylor[3,4,17], Steven A Enkemann[5,17], Ming-Sound Tsao[6,17], Timothy J Yeatman[5,17], William L Gerald[7,17], Steven Eschrich[5,17], Igor Jurisica[6,17], Thomas J Giordano[8], David E Misek[3,9], Andrew C Chang[3,9], Chang Qi Zhu[6], Daniel Strumpf[6], Samir Hanash[3], Frances A Shepherd[6], Keyue Ding[10], Lesley Seymour[10], Katsuhiko Naoki[11], Nathan Pennell[11], Barbara Weir[11], Roel Verhaak[11], Christine Ladd-Acosta[12], Todd Golub[12], Michael Gruidl[5], Anupama Sharma[5], Janos Szoke[7], Maureen Zakowski[7], Valerie Rusch[7], Mark Kris[7], Agnes Viale[7], Noriko Motoi[7], William Travis[7], Barbara Conley[13], Venkatraman E Seshan[14,17], Matthew Meyerson[11,12,17], Rork Kuick[3,17], Kevin K Dobbin[15,17], Tracy Lively[16,17], James W Jacobson[16,17] & David G Beer[3,9,17]

- Nat Med. 2008 Aug;14(8):822-7.
- Consists of four independent datasets:
  - – HLM – 79 tumor profiles
  - – Michigan - 177 tumor profiles
  - – Canada – 82 tumor profiles
  - – MSKCC – 104 tumor profiles
- Outcome is overall survival or progression-free survival

Cox correlation

Overall Survival

	Beta	P-value
HLM	0.014876	0.684682
MICH	0.02412	0.420761
Canada	0.117523	0.022689
MSKCC	0.071145	0.090277

Progression-free survival

	Beta	P-value
HLM	0.082129	0.054408
MICH	-0.002233	0.948287
Canada	0.0593	0.207077
MSKCC	0.08017	0.044657

- As described in the main paper, computed metastasis signature R-value for each tumor in each of the four datasets
  - *Here, metastasis signature did not include any of the GO or Whitfield cell cycle genes*
- Correlation of R-value with outcome across tumors measured by Cox

HLM

Statistically significant

Overall Survival

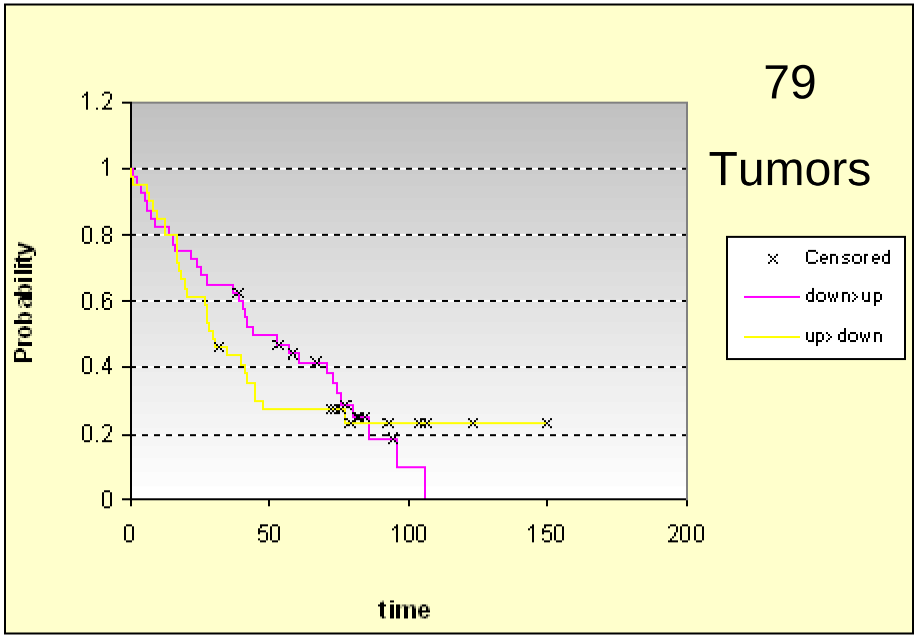

Chi-square	Degrees of Freedom	P
0.127050289	1	0.721510223

	Beta	P-value
HLM	0.014876	0.684682

Progression-free survival

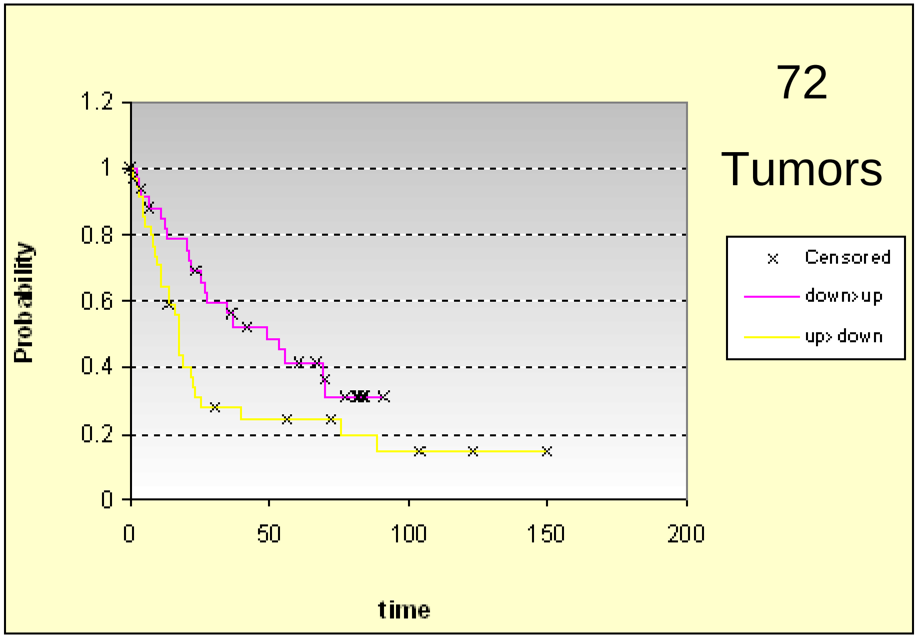

Chi-square	Degrees of Freedom	P
4.338550546	1	0.037258637

	Beta	P-value
HLM	0.082129	0.054408

- Compare tumors with high correlation to the signature (R-value>0) with the rest of the tumors by Kaplan-Meier
- Yellow = met signature positive, Pink = met signature negative

MICH **Not at all significant**

Overall Survival

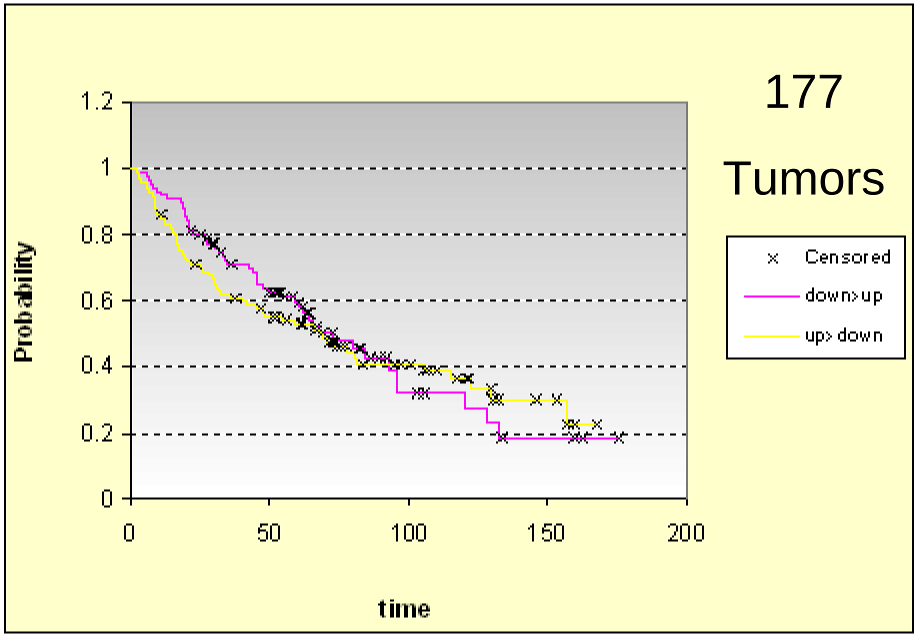

Chi-square	Degrees of Freedom	P
0.112141728	1	0.737718861

	Beta	P-value
MICH	0.02412	0.420761

Progression-free survival

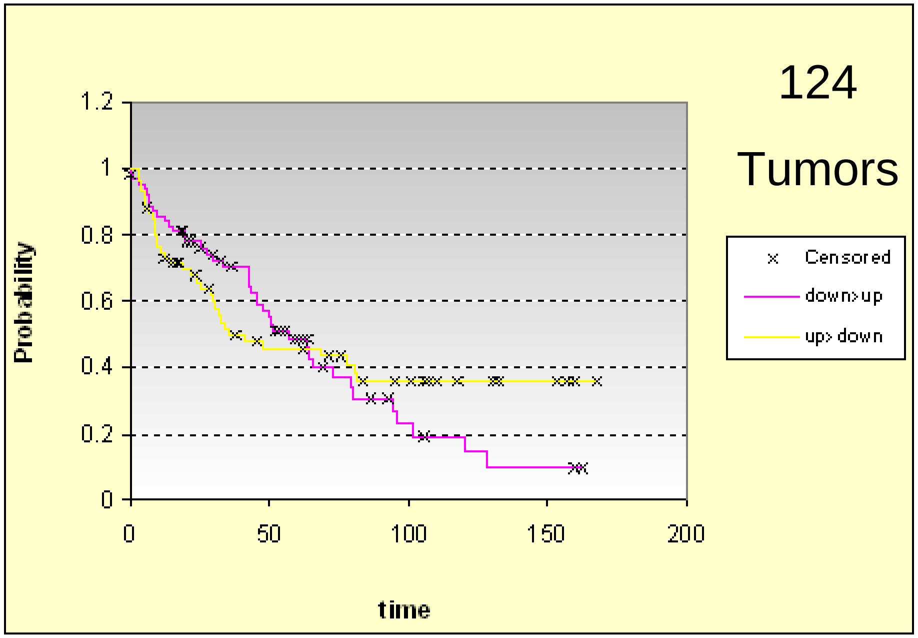

Chi-square	Degrees of Freedom	P
0.124882488	1	0.723798208

	Beta	P-value
MICH	-0.002233	0.948287

- Compare tumors with high correlation to the signature (R-value>0) with the rest of the tumors by Kaplan-Meier
- Yellow = met signature positive, Pink = met signature negative

Canada

Not significant, but perhaps trending

Overall Survival

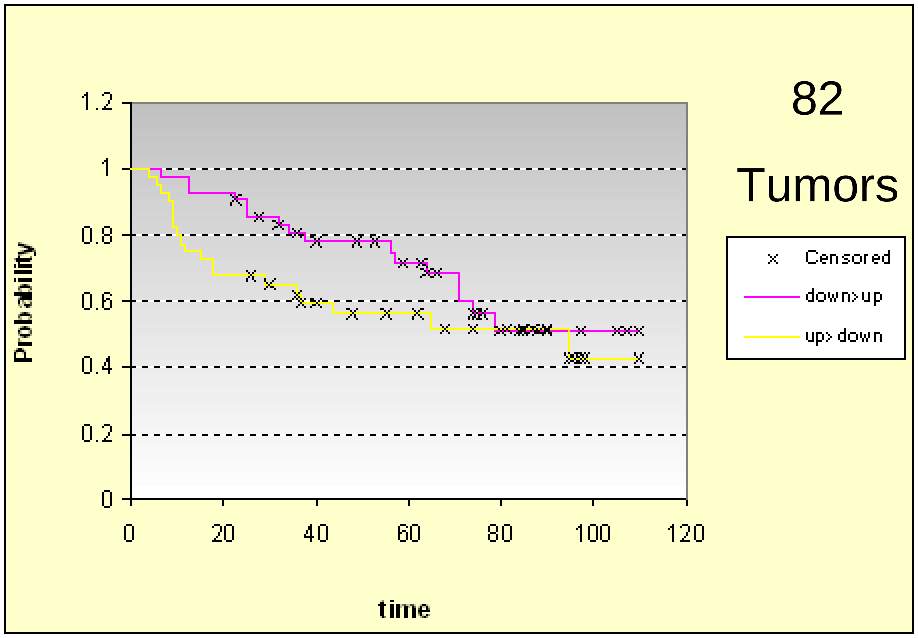

Chi-square	Degrees of Freedom	P
1.711693449	1	0.190765809

	Beta	P-value
Canada	0.117523	0.022689

Progression-free survival

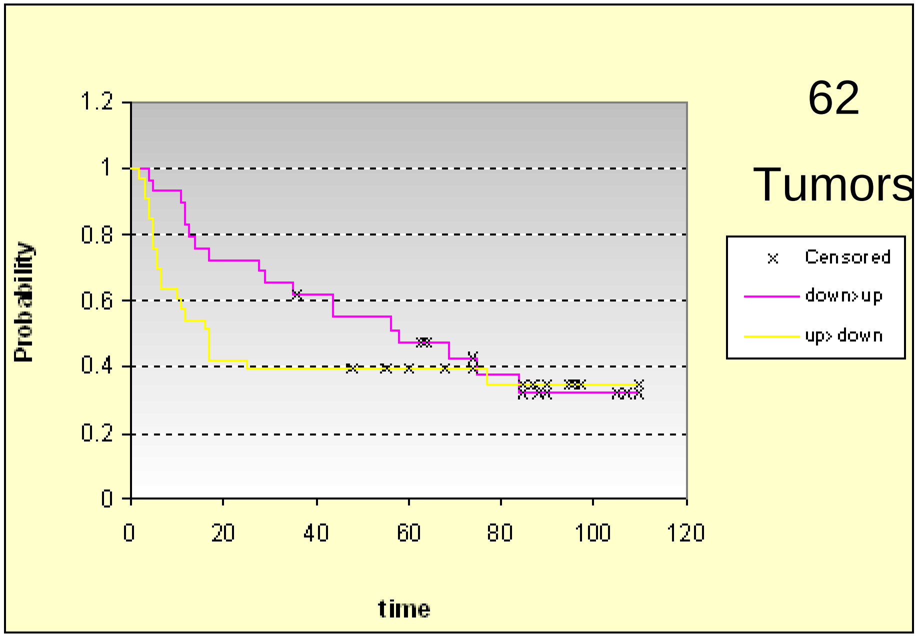

Chi-square	Degrees of Freedom	P
1.009555914	1	0.315009253

	Beta	P-value
Canada	0.0593	0.207077

- Compare tumors with high correlation to the signature (R-value>0) with the rest of the tumors by Kaplan-Meier
- Yellow = met signature positive, Pink = met signature negative

MSKCC

Statistically significant

Overall Survival

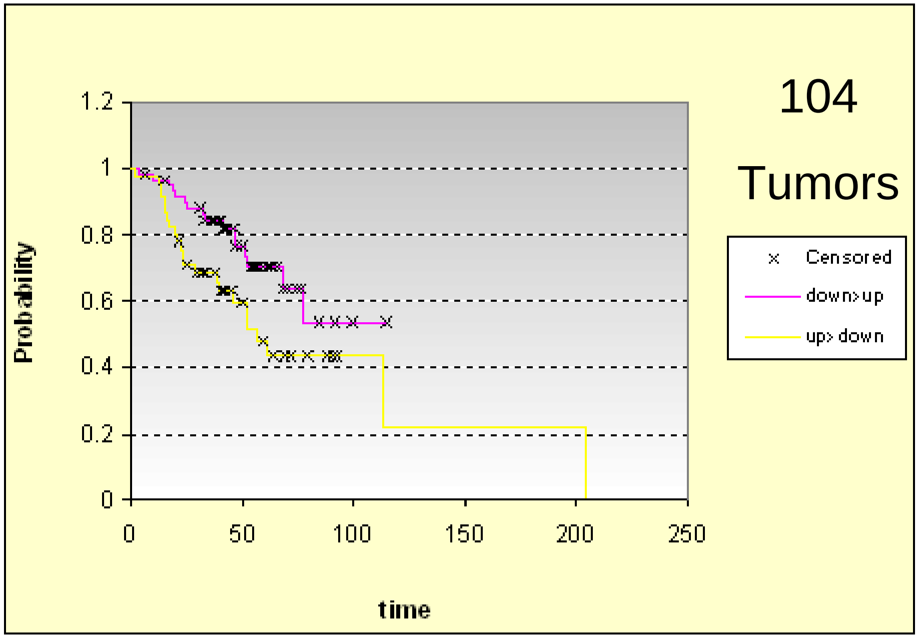

Chi-square	Degrees of Freedom	P
4.645796394	1	0.031129693

	Beta	P-value
MSKCC	0.071145	0.090277

Progression-free survival

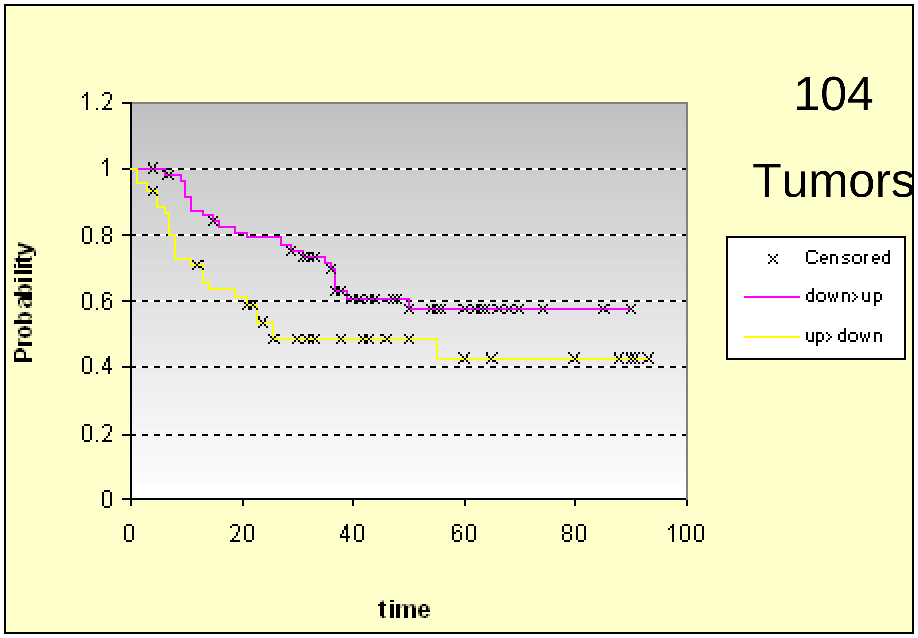

Chi-square	Degrees of Freedom	P
4.460642666	1	0.034684451

	Beta	P-value
MSKCC	0.08017	0.044657

- Compare tumors with high correlation to the signature (R-value>0) with the rest of the tumors by Kaplan-Meier
- Yellow = met signature positive, Pink = met signature negative

All 4 datasets

Statistically significant

Overall Survival

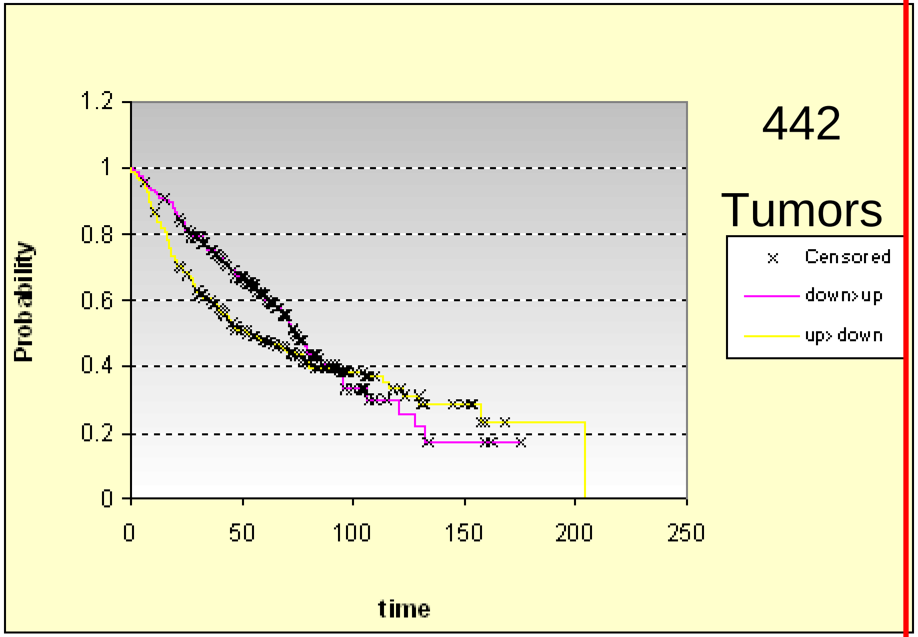

Chi-square	Degrees of Freedom	P
3.311187859	1	0.068809756

Progression-free survival

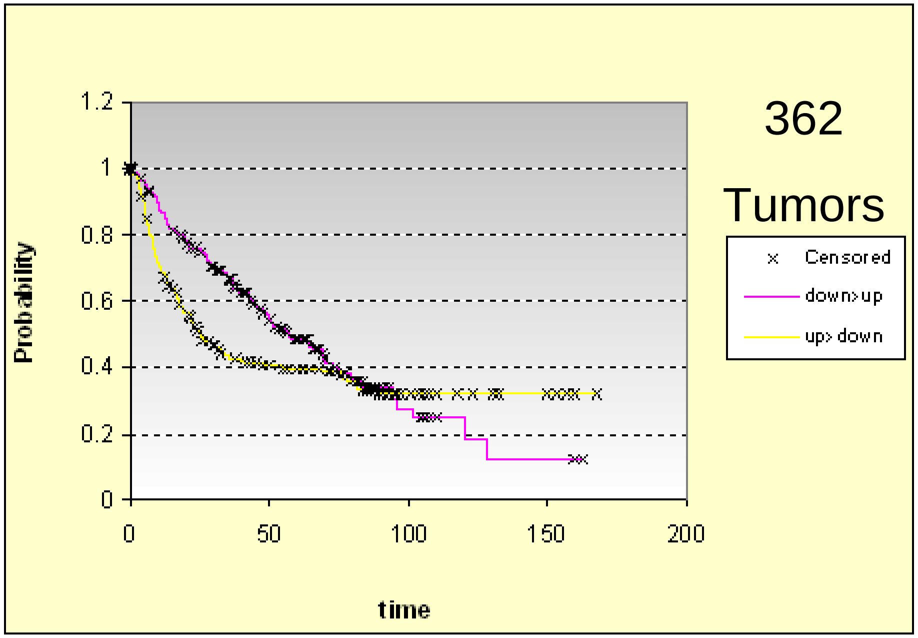

Chi-square	Degrees of Freedom	P
4.785514029	1	0.028700083

- Compare tumors with high correlation to the signature (R-value>0) with the rest of the tumors by Kaplan-Meier
- Yellow = met signature positive, Pink = met signature negative
- All 442 tumors from all four dataset included, P-value still nominal.

Conclusions

- Don’t see as strong a correlation with **overall survival** for the met signature with DC lung profile datasets
  - See trend for Canada and MSKCC datasets
- See a better correlation with **progression-free survival** for the met signature with DC lung profile datasets
