# Supplementary material for: Expression Signatures of Metastatic Capacity in a Genetic Mouse Model of Lung Adenocarcinoma
Source: PLoS One. 2009 Apr 30;4(4):e5401. doi: 10.1371/journal.pone.0005401 (PMC2671160; doi:10.1371/journal.pone.0005401)
Supplement: Figure S4 — PDF describing “Comparison of mouse metastasis signature with other metastasis signatures” (0.27 MB PDF) [file pone.0005401.s004.pdf]

Expression Signatures of Metastatic Capacity in a Genetic Mouse Model of Lung Adenocarcinoma

Supporting Document 2

(Comparison of mouse metastasis signature with other metastasis signatures)

Multiclass cancer diagnosis using tumor gene expression signatures

Sridhar Ramaswamy[*,†], Pablo Tamayo[*], Ryan Rifkin[*,‡], Sayan Mukherjee[*,‡], Chen-Hsiang Yeang[*,§], Michael Angelo[*], Christine Ladd[*], Michael Reich[*], Eva Latulippe[¶], Jill P. Mesirov[*], Tomaso Poggio[‡], William Gerald[¶], Massimo Loda[†,‖], Eric S. Lander[*,**], and Todd R. Golub[*,††,‡‡]

*Whitehead Institute/Massachusetts Institute of Technology Center for Genome Research, Cambridge, MA 02138; Departments of †Adult and ††Pediatric Oncology, Dana–Farber Cancer Institute/Harvard Medical School, Boston, MA 02115; ‖Department of Pathology, Brigham and Women's Hospital, Boston, MA 02115; ¶Department of Pathology, Memorial Sloan–Kettering Cancer Center, New York, NY 10021; and Departments of **Biology, ‡McGovern Institute, Center for Brain and Computational Learning, and §Artificial Intelligence Laboratory, Massachusetts Institute of Technology, Cambridge, MA 02139

Contributed by Eric S. Lander, October 23, 2001

- "Global Cancer Map" (GCM) of multiple tumor types, including metastases
- PNAS, 2001 Dec 18;98(26):15149-54.
- Compare human metastasis-associated genes with mouse model metastasis-associated genes

Intersection of genes both high in k-ras/p53mut mouse metastasis and genes high in human tumor metastases

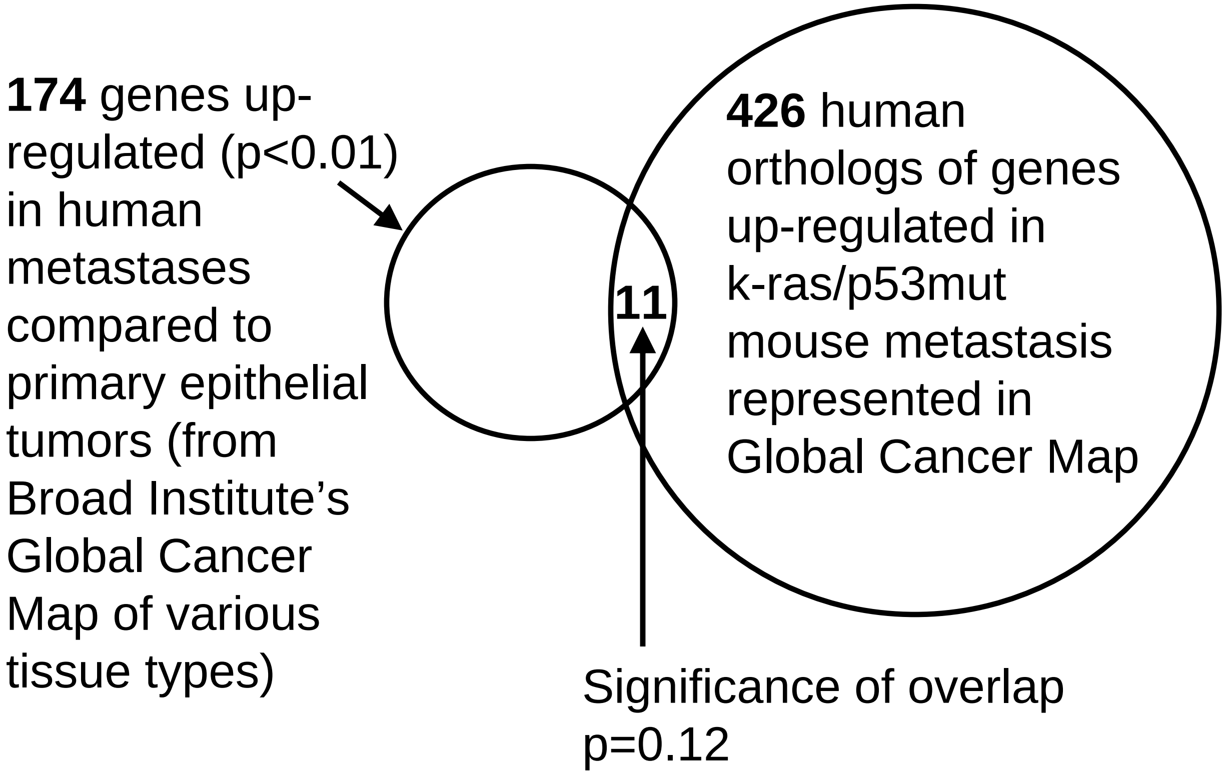

(10052 genes represented in GCM dataset.)

If we relax statistical cutoffs, do see significant overlap between human and mouse systems

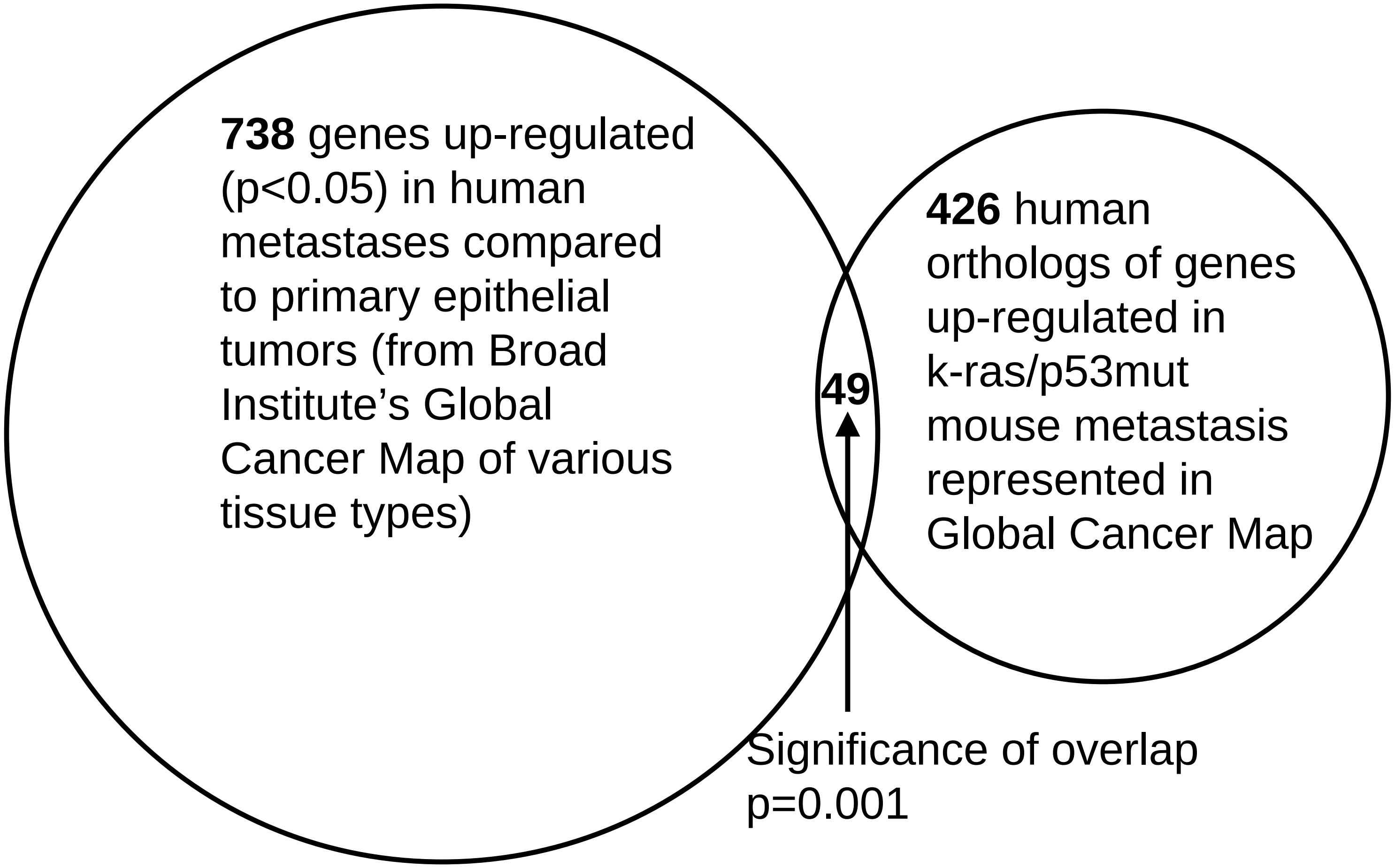

(10052 genes represented in GCM dataset.)

Entrez ID	Name	Title
1278	COL1A2	collagen, type I, alpha 2
94239	H2AFV	H2A histone family, member V
2199	FBLN2	fibulin 2
4085	MAD2L1	MAD2 mitotic arrest deficient-like 1 (yeast)
2146	EZH2	enhancer of zeste homolog 2 (Drosophila)
6624	FSCN1	fascin homolog 1, actin-bundling protein (Strongylocentrotus purpuratus)
4605	MYBL2	v-myb myeloblastosis viral oncogene homolog (avian)-like 2
1277	COL1A1	collagen, type I, alpha 1
1545	CYP1B1	cytochrome P450, family 1, subfamily B, polypeptide 1
8880	FUBP1	Far upstream element (FUSE) binding protein 1
79101	JOSD3	Josephin domain containing 3
388753	C1orf31	chromosome 1 open reading frame 31
1725	DHPS	deoxyhypusine synthase
2000	ELF4	E74-like factor 4 (ets domain transcription factor)
2305	FOXM1	forkhead box M1
3181	HNRPA2B1	heterogeneous nuclear ribonucleoprotein A2/B1
3192	HNRPU	heterogeneous nuclear ribonucleoprotein U (scaffold attachment factor A)
4591	TRIM37	tripartite motif-containing 37
11167	FSTL1	follistatin-like 1
10531	PITRM1	pitrilysin metallopeptidase 1
27258	LSM3	LSM3 homolog, U6 small nuclear RNA associated (S. cerevisiae)
2113	ETS1	v-ets erythroblastosis virus E26 oncogene homolog 1 (avian)
3998	LMAN1	Lectin, mannose-binding, 1
3035	HARS	histidyl-tRNA synthetase
10658	CUGBP1	CUG triplet repeat, RNA binding protein 1
332	BIRC5	baculoviral IAP repeat-containing 5 (survivin)
9055	PRC1	protein regulator of cytokinesis 1
10411	RAPGEF3	Rap guanine nucleotide exchange factor (GEF) 3
56262	LRRC8A	leucine rich repeat containing 8 family, member A
28989	C9orf32	chromosome 9 open reading frame 32
3609	ILF3	interleukin enhancer binding factor 3, 90kDa
5176	SERPINF1	serpin peptidase inhibitor, clade F (alpha-2 antiplasmin, pigment epithelium derived factor)
87178	PNPT1	polyribonucleotide nucleotidyltransferase 1
7070	THY1	Thy-1 cell surface antigen
8078	USP5	ubiquitin specific peptidase 5 (isopeptidase T)
9564	BCAR1	breast cancer anti-estrogen resistance 1
6118	RPA2	replication protein A2, 32kDa
10432	RBM14	RNA binding motif protein 14
56943	ENY2	enhancer of yellow 2 homolog (Drosophila)
1281	COL3A1	collagen, type III, alpha 1 (Ehlers-Danlos syndrome type IV, autosomal dominant)
8565	YARS	tyrosyl-tRNA synthetase
92400	RBM18	RNA binding motif protein 18
1786	DNMT1	DNA (cytosine-5-)-methyltransferase 1
94104	C21orf66	chromosome 21 open reading frame 66
4150	MAZ	MYC-associated zinc finger protein (purine-binding transcription factor)
11004	KIF2C	kinesin family member 2C
5984	RFC4	replication factor C (activator 1) 4, 37kDa
8239	USP9X	ubiquitin specific peptidase 9, X-linked
1164	CKS2	CDC28 protein kinase regulatory subunit 2

- The list of 49 genes that were found high in both the mouse metastasis signature ($P<0.01$, paired t-test) and high in metastasis versus primary samples ($P<0.05$, unpaired t-test)

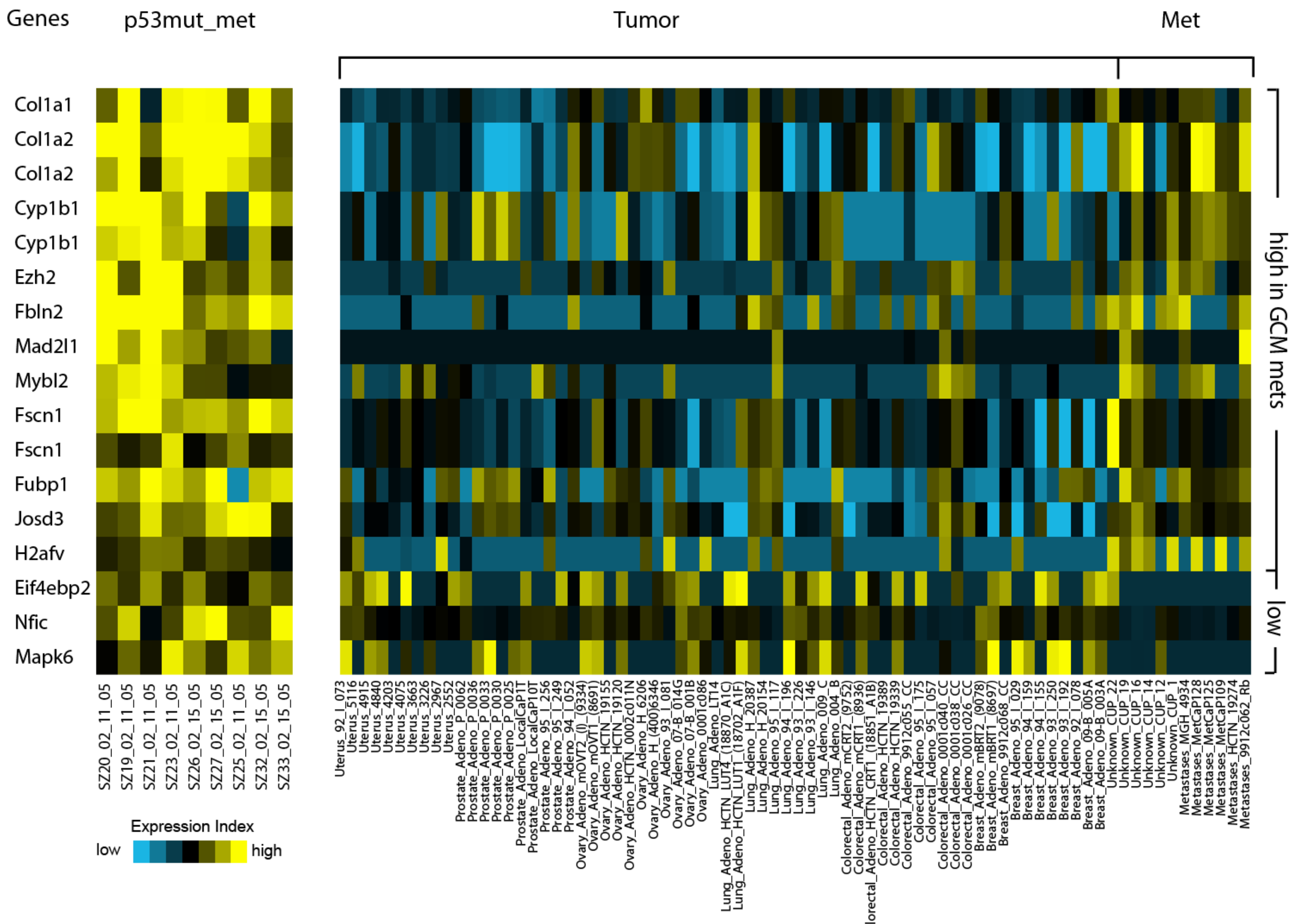

- Genes that were both high (p<0.01) in metastases from p53 mutant mice and differentially expressed (p<0.01) in human metastases compared to primary tumors (various histological types).

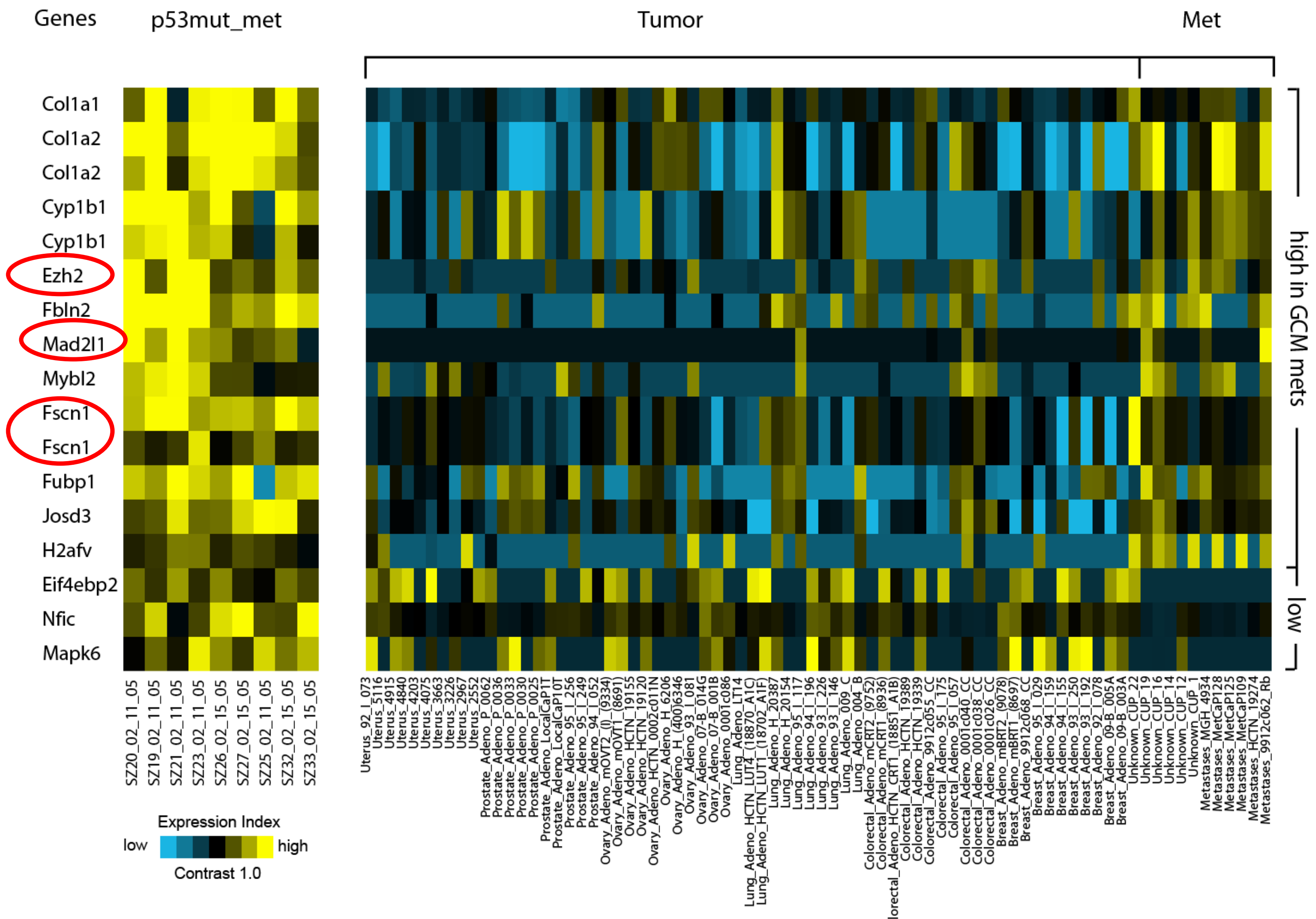

- Indicated genes were also correlated (p<0.05) with poor prognosis in profiles of human primary lung tumors (from dataset by Bhattacharjee *et al.*).

Conclusions

- Some degree of significant overlap between genes high in human metastasis versus primary tumors and genes high in our mouse metastasis model.
  - Unable to do a paired analysis in the human dataset, but we cannot tell whether that would be an issue here as to why the overlap was not more significant
- Overlap not significant for the genes *low* in metastasis (results not shown).
